# Evaluating a smartphone digits-in-noise test as part of the audiometric test battery

**DOI:** 10.4102/sajcd.v65i1.574

**Published:** 2018-05-21

**Authors:** Jenni-Mari Potgieter, De Wet Swanepoel, Cas Smits

**Affiliations:** 1 Department of Speech-Language Pathology and Audiology, University of Pretoria, South Africa; 2 Ear Sciences Centre, School of Surgery, University of Western Australia, Ear Science Institute Australia, Australia; 3 Department of Otolaryngology, Head and Neck Surgery, Section Ear and Hearing, the Netherlands; 4 Amsterdam Public Health Research Institute, VU University Medical Center, the Netherlands

## Abstract

**Background:**

Speech-in-noise tests have become a valuable part of the audiometric test battery providing an indication of a listener’s ability to function in background noise. A simple digits-in-noise (DIN) test could be valuable to support diagnostic hearing assessments, hearing aid fittings and counselling for both paediatric and adult populations.

**Objective:**

The objective of this study was to evaluate the South African English smartphone DIN test’s performance as part of the audiometric test battery.

**Design:**

This descriptive study evaluated 109 adult subjects (43 male and 66 female subjects) with and without sensorineural hearing loss by comparing pure-tone air conduction thresholds, speech recognition monaural performance scores (SRS dB) and the DIN speech reception threshold (SRT). An additional nine adult hearing aid users (four male and five female subjects) were included in a subset to determine aided and unaided DIN SRTs.

**Results:**

The DIN SRT is strongly associated with the best ear 4 frequency pure-tone average (4FPTA) (*r_s_* = 0.81) and maximum SRS dB (*r* = 0.72). The DIN test had high sensitivity and specificity to identify abnormal pure-tone (0.88 and 0.88, respectively) and SRS dB (0.76 and 0.88, respectively) results. There was a mean signal-to-noise ratio (SNR) improvement in the aided condition that demonstrated an overall benefit of 0.84 SNR dB.

**Conclusion:**

The DIN SRT was significantly correlated with the best ear 4FPTA and maximum SRS dB. The DIN SRT provides a useful measure of speech recognition in noise that can evaluate hearing aid fittings, manage counselling and hearing expectations.

## Introduction

### Background

One of the major problems persons with hearing loss experience is communication in the presence of background noise (Taylor, 2003). Amplification options such as hearing aids, cochlear implants and assistive listening devices could improve hearing abilities, but most hearing-impaired listeners still find it difficult to understand conversation in background noise (Smits, Goverts, & Festen, 2013). Speech-in-noise tests have become an important asset to the diagnostic audiometric test battery as pure-tone air conduction testing and speech recognition scores are not able to determine or mimic the everyday challenge of listening to speech-in-noise (Taylor, 2003).

By the late 1970s, speech-in-noise tests became popular as a result of the pioneering work by Plomp and Mimpen (1979) with the development of the standard Dutch speech-in-noise test. The standard Dutch speech-in-noise test was able to reliably determine the speech reception threshold (SRT) for sentences. Many variations of the standard Dutch speech-in-noise test were developed in several languages because of the test’s strong validity, reliability, sensitivity and specificity (Plomp & Mimpen, 1979; Theunissen, Swanepoel, & Hanekom, 2009). Examples of such tests include the HINT (Hearing In Noise Test), MHINT (Mandarin Hearing In Noise Test) and the German sentence test (Kollmeier & Wesselkamp, 1997; Nilsson, Soli & Sullivan, 1994; Wong, Soli, Liu, Han, & Huang, 2007).

Today speech-in-noise tests are primarily used in the clinical setting to determine a person’s ability to function in a general communication environment by evaluating the speech understanding handicap caused by the hearing loss (Smits et al., 2013). Speech-in-noise tests possess additional clinical value because information on speech understanding in noise can support adjustment and monitoring of hearing aid and cochlear implant fitting parameters (Smits et al., 2013). Speech-in-noise tests have also played an important role in counselling hearing aid or cochlear implant users to understand their hearing disability, manage expectations and implement intervention approaches (Kaandorp, Smits, Merkus, Goverts, & Festen, 2015; Smits et al., 2013).

Most speech-in-noise tests use meaningful sentences as speech material because sentences are representative of daily conversation. Even though speech-in-noise sentence tests are able to determine hearing loss for speech, the appropriateness of such a test may be limited (Smits et al., 2013; Theunissen et al., 2009). To administer a speech-in-noise sentence test, the listener must be able to understand a whole sentence correctly at a comfortable signal-to-noise ratio (SNR). Most listeners with a hearing loss perform poorly on speech recognition tasks as a result of the severity of the hearing loss or language competence, especially in additional language listeners (Potgieter, Swanepoel, Myburg, & Smits, 2017; Smits et al., 2013). Therefore, listeners with poor additional language competence, cochlear implant users, children and people with severe hearing losses are not able to undertake a speech-in-noise sentence test (Potgieter et al., 2017; Smits et al., 2013).

More recently the digits-in-noise test (DIN) was developed for diagnostic and clinical purposes (Smits et al., 2013). This test, using digit-triplet (i.e. 1-6-5) material, was demonstrated to be suitable as speech material in a diagnostic DIN by comparing it to the gold standard Dutch sentence test developed by Plomp and Mimpen (1979). This study concluded that the digit-triplets test demonstrated no learning effect and an accurate SRT could be determined (Smits et al., 2013). The DIN also had high criterion validity, and the steepness of the slope for the speech recognition function compared positively to the Dutch sentence test (Smits et al., 2013). Additionally, the DIN test could be conducted from normal to profound hearing losses. The simplicity of the DIN test even allows children to conduct the test (Smits et al., 2013).

Because of the successful development of the DIN, the South African smartphone-based DIN hearing test was developed in 2016 (Potgieter, Swanepoel, Myburgh, Hopper, & Smits, 2016; Smits et al., 2013). Following a similar development and validation procedure as the Dutch DIN test (Potgieter et al., 2016; Smits et al., 2013), the South African DIN was developed using South African English digits (0–9) as speech material. The noise level was fixed for negative SNRs, whereas the speech was fixed for positive SNRs. An adaptive test procedure was followed where a triplet was presented 2 dB higher (correct response) or 2 dB lower (incorrect response) based on the subject’s response. The SRT was calculated as the average SNR of the triplets presented. A cut-off value was determined at -9.55 dB to indicate ‘pass’ or ‘refer’ for hearing loss in native English speakers or non-native speakers with a high level of self-reported English-speaking competence (Potgieter et al., 2017; Smits et al., 2013).

Potgieter et al. (2017) demonstrated that the South African DIN could accommodate non-native listeners by adjusting the ‘pass’ or ‘refer’ criteria based on self-reported English-speaking competency. The South African DIN, therefore, ensures an accurate test result across native and non-native South African English listeners (Potgieter et al., 2017). Additionally, the increased use of smartphones in South Africa allows the DIN to be available to increasing numbers of South Africans living in rural and urban areas (Potgieter et al., 2016, 2017).

Based on the successful implementation of the South African DIN as a screening test [available on a smartphone application (App)], its suitability for clinical use in an audiology clinic required investigation. Smits et al. (2013) reported that the diagnostic DIN test could be an important asset to the audiometric test battery for the following two reasons: Firstly, in South Africa, no standardised or validated recorded speech materials for spondee or phonetically balanced word lists exists. A diagnostic version of the South African DIN can provide additional information on a listener’s hearing impairment for speech recognition in noise. The DIN is validated and consists of recorded speech material with low linguistic demands suitable to test normal hearing to profound hearing losses (Potgieter et al., 2016; Smits et al., 2013). Secondly, the test could assist in counselling and management of a listener’s hearing aid expectation as well as assessing hearing aid benefit (Smits et al., 2013).

### Objective

Given the potential benefit of a diagnostic version of the South African DIN, this study aimed to compare the DIN alongside standard diagnostic audiology tests in clinical practice. A comparison was conducted between pure-tone audiometry, speech recognition monaural performance scores (SRS dB) and the DIN SRT. The DIN was also used in a subset of participants to explore the potential benefit for use with hearing aid listeners by determining aided and unaided DIN SRTs.

## Methodology

The institutional review board of the University of Pretoria approved the research study before data collection commenced.

### Subjects

A comparative and correlational descriptive research design was followed. Various audiometric practices in the Gauteng region assisted with data collection. Each audiologist was supplied with a smartphone to conduct the South African English smartphone-based DIN on subjects in their own private practice or at the public health hospital audiology clinic. A convenience non-probability sampling procedure was followed with selected subjects as they were available and willing to volunteer to take part in the research study at clinical data collection sites. The subjects were assessed with a comprehensive audiometric test battery and the smartphone DIN in a single test session lasting approximately 1 h.

### Methods and materials

An otoscopic evaluation was performed to allow observation of any obstruction in the external auditory meatus. Any ear canal obstructions were removed by a qualified audiologist or healthcare provider before testing commenced.

A variety of clinical audiometers were used to conduct pure-tone air conduction, bone conduction and speech recognition testing. Air conduction and speech audiometry were done in a soundproof booth using supra-aural or insert earphones. A bone oscillator was used to conduct bone conduction audiometry. Air conduction thresholds across octave frequencies from 250 to 8000 Hz were determined, while bone conduction thresholds were determined from 500 to 4000 Hz. The modified Hughson–Westlake method was used to seek pure-tone air and bone conduction thresholds (Hughson & Westlake, 1944). The pure-tone average (500, 1000, 2000 and 4000 Hz) was calculated to categorise the severity and configuration of the hearing impairment according to the Jerger criteria (Jerger & Jerger, 1980). Normal-hearing was categorised as normal if the best ear 4 frequency pure-tone average (4FPTA) was ≤25 dB HL. A mixed hearing loss was determined by calculating the pure-tone and bone conduction averages (500, 1000, 2000 and 4000 Hz) for both ears. The hearing loss was categorised as conductive or mixed when the average threshold difference was >15 dB HL (Margolis & Saly, 2007).

Speech recognition testing was presented with live voice in Afrikaans or English. Speech recognition monaural performance scores were obtained across intensities by administering the *Afrikaanse Foneties Gebalanseerde Woordelys* (a phonetically balanced word list) to Afrikaans-speaking subjects (Laubscher & Tesner, 1966). The *University of Pretoria, English Phonetically Balanced Word List* was used to obtain SRS dB in first-language English speakers. A list of 25 phonetically balanced words was presented 30 dB HL above the 4FPTA at three different intensities (maximum intensity 90 dB). English additional language speakers could choose whether they would like speech recognition testing to be presented in Afrikaans or English. Normal maximum SRS dB was classified as a 100% word discrimination score at intensities ≤40 dB HL. The best ear maximum SRS dB was used in the analysis. The 50% SRS dB was not available for all subjects. Various sites were involved in data collection, thereby not using the same methods for determining SRS dB scores.

The South African smartphone DIN App instructed the subject to enter his or her gender, date of birth, initials and surname. The subject placed the smartphone earphone set into the ears and listened to digits being repeated. A scroll bar allowed the subject to adjust the volume of the digits being repeated to a comfortable listening intensity. A ‘start test’ button presented the test. The subject entered the digit responses on the smartphone keypad.

Once the DIN hearing screening test started, the test operates by varying the noise intensity level while having a fixed speech level when triplets with negative SNRs are presented. When triplets with positive SNRs are presented, the speech level becomes fixed and the noise level varies. The noise starts 500 ms before triplet presentation and stops 500 ms after triplet presentation. The digits were pronounced by a female speaker with natural intonation, for example, 6–9–0, spoken as six–nine–zero. The first digit-triplet set was presented at the subject’s comfortable listening intensity. The subject responded to the triplet set by entering the digit-triplet set on a pop-up keypad. The next digit is presented 2 dB higher (incorrect response) or 2 dB lower (correct response) based on the subject’s response.

A subgroup of nine hearing aid users were asked to perform an additional DIN using their hearing aids. The DIN was presented using free-field speakers in a soundproof booth. The smartphone were connected to the free-field speakers. The subjects were seated 1 m from the speaker facing the speaker at 0°. The DIN was operated using the smartphone App and the subjects entered their response using the smartphone App.

## Data analysis

A Spearman correlation coefficient was determined to assess the relationship between the best ear 4FPTA, maximum SRS dB and the DIN SRT (1% significance level used).

The performance of the South African English smartphone DIN was determined by comparing the SRT to the best ear 4FPTA and maximum SRS dB. Descriptive statistics, frequencies and proportions were determined for these variables. The sensitivity and specificity, positive predictive value (PPV) and negative predictive value (NPV) were calculated as an indication of the accuracy of the method for determining a hearing loss. Additionally, the area under the curve (AUROC) from a receiver operation characteristic curve (ROC) analysis was determined to provide a further indication of the accuracy between the variables.

Descriptive statistics for aided and unaided hearing aid DIN SRTs were determined for a subset of nine subjects.

## Results

A total of 109 adult subjects (43 male and 66 female subjects) participated in this study. The mean age was 55 years (20 SD) with a range of 16–89 years. The sample included 50 normal-hearing subjects and 59 subjects with a hearing loss. An additional subset of nine adult subjects (4 male and 5 female subjects) with an average age of 72 years (7.2 SD; 63–84 years range) participated in this sub-study.

### Comparing digits-in-noise speech reception threshold, pure-tone audiometry and maximum speech recognition monaural performance score intensity

**FIGURE 1:**
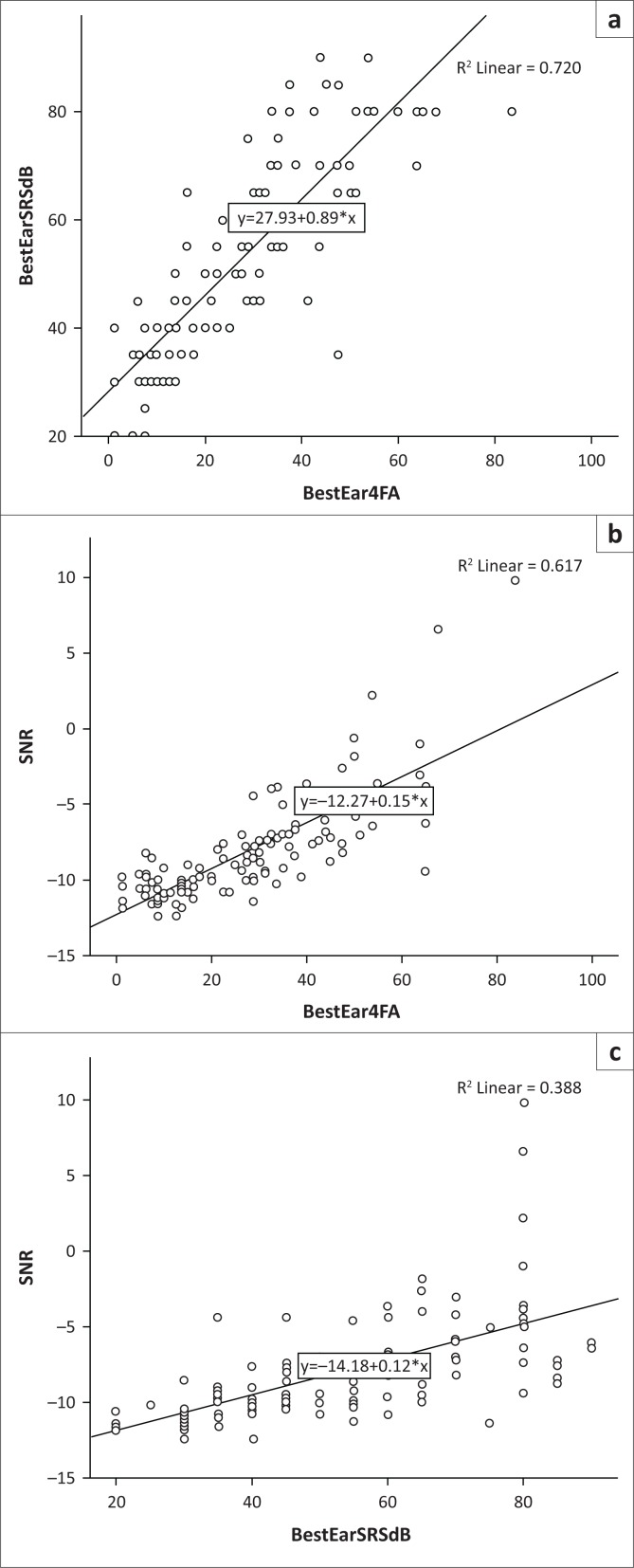
(a) Scatterplot indicating the correlation between the best ear 4 frequency pure-tone average and maximum speech recognition monaural performance scores; (b) Scatterplot indicating the correlation between the best ear 4 frequency pure-tone average and digits-in-noise signal-to-noise ratio; (c) Scatterplot indicating the correlation between the best ear maximum speech recognition monaural performance scores and digits-in-noise signal-to-noise ratio.

Significant correlations (Figure 1a) were evident between the best ear 4FPTA and maximum SRS dB (*r* = 0.87; *n* = 111; *p* < 0.001), and the best ear 4FPTA and DIN SRT (Figure 1b) (*r*_s_ = 0.81; *n* = 120; *p* < 0.001) and the maximum SRS dB and DIN SRT (Figure 1c) (*r* = 0.72; *n* = 111; *p* < 0.001). The strongest correlation was between the best ear 4FPTA and maximum SRS dB and the weakest correlation was between the maximum SRS dB and the DIN SRT. A comparison between the best ear 4FPTA (28.8 Mean; 17.9 SD) and SRS dB (53.6 Mean; 18.9 SD) showed a significant difference between the two variables.

High sensitivity and specificity were obtained when comparing the DIN SRT to the maximum SRS dB HL (Table 1). The PPV of the DIN SRT was 91.5% and NPV was 66% to identify subjects with and without an abnormal maximum SRS dB result (≤100% word discrimination score at ≥40 dB HL).

**TABLE 1:** Performance of digits-in-noise speech reception threshold and maximum speech recognition monaural performance scores to predict normal and abnormal best ear 4 frequency pure-tone average (SNR cut-off = −9.5 dB SNR).

Variable	HL (*n*)	NH (*n*)	Sens	Spec	AUROC
BE^a^ SRT predicting BE 4FPTA	50	59	0.883	0.878	0.941
Maximum SRS dB predicting BE 4FPTA	60	49	0.831	0.974	0.937
BE SRT predicting BE SRS dB	59	50	0.76	0.868	0.884

SNR, signal-to-noise ratio; SRT, speech reception threshold; SRS dB, speech recognition monaural performance scores; 4FPTA, 4 frequency pure-tone average; dB, decibel; BE, best ear; Sens, sensitivity; Spec, specificity; AUROC, area under the curve; HL, hearing loss; NH, normal-hearing.

The DIN SRT had a high sensitivity and specificity to identify subjects with an abnormal 4FPTA result (Table 1). The PPV of the DIN SRT was 89.8% and NPV was 86% to identify subjects with and without an abnormal 4FPTA result.

The best ear maximum SRS dB predicted normal and abnormal best ear 4FPTA, with a high sensitivity and specificity (Table 1). The PPV of the maximum SRS dB HL was 98.3% and the NPV was 75.5% to identify subjects with and without an abnormal 4FPTA result.

A comparison between aided hearing aid DIN SRTs (−7.2 Mean; 2.1 SD; −3.2 to −9.4 range) and unaided hearing aid DIN SRTs (−6.4 Mean; 2.6 SD; −2 to −9.4 range) showed a small increase in SRT with hearing aids (0.8 Mean; 1.5 SD). There was significant individual variability between subjects in the aided condition (−3.2 to −9.4 range) and unaided condition (−2 to −9.4 range) (Table 2).

**TABLE 2:** Descriptive statistics for aided and unaided hearing aid digits-in-noise speech reception thresholds (*n* = 9).

Variable	Mean	Range	Standard deviation
Age	72	63 to 84	7.2
Best ear 4FPTA	37.6	26.2 to 47.5	8.1
Digits-in-noise SRT (aided)	−7.2	−9.4 to −3.2	2.1
Digits-in-noise SRT (unaided)	−6.3	−9.4 to −2	2.6
Digits-in-noise SRT difference	−0.84	−3.6 to 1.6	1.5

SRT, speech reception threshold; 4PETA, 4 frequency pure-tone average.

## Discussion

An English smartphone DIN was successfully developed in 2016 to provide widespread hearing screening in South Africa (Potgieter et al., 2016). Smits et al. (2013) implemented a diagnostic version of the DIN to support the diagnostic audiometric test battery in determining the speech recognition impairment in noise for listeners (Smits et al., 2013). In order to determine the South African English DIN hearing test’s applicability as a diagnostic tool, the DIN SRT was compared to the audiometric 4FPTA (best ear) and maximum SRS dB (best ear) in the current study.

The smartphone DIN SRT and the best ear 4FPTA was significantly correlated (*r*_s_ = 0.81) in line with previous results reported for the Dutch (*r* = 0.72), French (*r* = 0.77) and American English (*r* = 0.74) landline telephone DIN hearing screening tests (Jansen, Luts, Wagener, Frachet, & Wouters, 2010; Smits, Kapteyn, & Houtgast, 2004; Watson, Kidd, Miller, Smits, & Humes, 2012). A good relationship between the DIN SRT and maximum SRS dB was demonstrated (*r*_s_ = 0.72) and corresponded to previous results comparing the Northwestern University Auditory Test No. 6 in quiet to the Words-in-Noise Test (*R*s = 0.61) (Wilson, 2011). The AUROC for the DIN SRT and best ear 4FPTA comparison (0.941) in this study compared well to the AUROC for the Dutch (0.974) landline DIN (Smits et al., 2004).

The sensitivity and specificity of the DIN provides an indication of the DIN SRT’s ability to correctly identify listeners with or without a hearing loss. The sensitivity and specificity of the DIN SRT compared to the best ear 4FPTA (0.88 and 0.88, respectively) related well to the Dutch (0.91 and 0.93, respectively) and American English (0.80 and 0.83, respectively) landline DINs (Smits et al., 2004; Watson et al., 2012). A high sensitivity (0.76) and specificity (0.87) were also found when comparing the DIN SRT to the maximum SRS dB. The poorest correlation was between the DIN SRT and maximum SRS dB. Unsurprisingly, these tests are very different in what they measure with the SRS dB being a supra-threshold speech test in quiet while the DIN is a threshold test in noise (Lucks Mendel, 2008; Taylor, 2003; Wilson, 2011). The tests therefore complement each other within a clinical test battery. The SRS dB would always remain a fundamental part of the audiometric test battery as it is a method to crosscheck the pure-tone threshold and provides information on speech processing, sensitivity to speech stimuli and understanding speech at supra-threshold levels in quiet (Lucks Mendel, 2008). The DIN reflects a person’s speech recognition ability in noise and provides an indication of loss for speech-in-noise ability (Smits et al., 2013; Taylor, 2003; Wilson, 2011). The DIN can therefore inform counselling and hearing aid expectation management (Smits et al., 2013; Taylor, 2003; Wilson, 2011).

Pure-tone threshold testing forms an essential part of the audiometric test battery as the measurement provides information regarding a listener’s degree, type and configuration of hearing loss (Kramer, Kapteyn, Festen, & Tobi, 1996). Pure-tone thresholds, however, are unable to provide insight into speech recognition abilities in background noise (Kramer et al., 1996; Smits et al., 2004). Speech-in-noise tests (i.e. the DIN) are most valuable in diagnosing a listener’s speech recognition impairment in noise (Smits et al., 2013; Taylor, 2003). The results from the comparison between the DIN SRT and best ear 4FPTA show a strong relationship between these measures. The DIN SRT is therefore strongly associated with the 4FPTA but provides complementary information on speech recognition impairment in noise. Additionally, the DIN can be an applicable asset to the diagnostic audiometric test battery for the following reasons. Firstly, the DIN is easy to administer and takes a few minutes to conduct (Potgieter et al., 2016). Secondly, simple speech material is used requiring low linguistic demands (Potgieter et al., 2016; Smits et al., 2004). Thirdly, the DIN can be conducted from normal-hearing to profound hearing losses. Fourthly, the test is user friendly and can be used to test children (Smits et al., 2013). Finally, non-native English listeners with poor English language–speaking competence are able to conduct the DIN (Potgieter et al., 2017).

A small sample (*n* = 9) of hearing aid users were evaluated with the DIN with and without hearing aid amplification. The mean SNR improved in the aided condition and demonstrated an overall benefit of 0.84 SNR dB. There was significant individual variability between subjects in the aided condition (−3.2 to −9.4 SNR dB) and unaided condition (−2 to −9.4 SNR dB). The DIN can be valuable in a clinical audiology setting to provide individualised performance measures for hearing aid users in background noise (Smits et al., 2013; Taylor, 2003). A measure of speech-in-noise ability is a valuable clinical addition for counselling and demonstrating hearing aid benefit in the presence of background noise (Smits et al., 2013; Taylor, 2003). Counselling informed by the DIN SRT could assist hearing aid users to understand their hearing impairment and provide important information regarding the communication difficulties that may persist (Smits et al., 2013; Taylor, 2003). The hearing aid could also be adjusted according to the hearing aid user’s needs as reflected on the DIN SRT (Taylor, 2003). The limitation of this study included the small sample of hearing aid users evaluated with and without hearing aid amplification using the DIN and the lack of data for 50% SRS dB scores.

## Conclusion

The DIN SRT is strongly associated with the best ear 4FPTA and maximum SRS dB and could therefore provide complementary information on speech recognition impairment in noise. The DIN had high sensitivity and specificity to identify abnormal pure-tone and SRS dB results. The DIN SRT and best ear 4FPTA were significantly correlated with previously developed landline telephone DINs. The DIN SRT can also demonstrate benefit for hearing aid fittings. The test is quick to administer, inexpensive, readily available and provides information on the SNR loss. The DIN SRT could therefore be used as a counselling tool to evaluate hearing aid fittings, manage counselling and hearing expectations.
